# Understanding and Reducing False Alarms in Observational Fog Prediction

**DOI:** 10.1007/s10546-018-0374-2

**Published:** 2018-07-03

**Authors:** Jonathan G. Izett, Bas J. H. van de Wiel, Peter Baas, Fred C. Bosveld

**Affiliations:** 1 Department of Geoscience and Remote Sensing, Delft University of Technology, Delft, The Netherlands; 2 Royal Netherlands Meteorological Institute (KNMI), De Bilt, The Netherlands

**Keywords:** Cabauw site, False alarms, Fog forecasting, Observations of fog, Radiation fog

## Abstract

The reduction in visibility that accompanies fog events presents a hazard to human safety and navigation. However, accurate fog prediction remains elusive, with numerical methods often unable to capture the conditions of fog formation, and observational methods having high false-alarm rates in order to obtain high hit rates of prediction. In this work, 5 years of observations from the Cabauw Experimental Site for Atmospheric Research are used to further investigate how false alarms may be reduced using the statistical method for diagnosing radiation-fog events from observations developed by Menut et al. (Boundary-Layer Meteorol 150:277–297, 2014). The method is assessed for forecast lead times of 1–6 h and implementing four optimization schemes to tune the prediction for different needs, compromising between confidence and risk. Prediction scores improve significantly with decreased lead time, with the possibility of achieving a hit rate of over 90% and a false-alarm rate of just 13%. In total, a further 31 combinations of predictive variables beyond the original combination are explored (including mostly, e.g., variables related to moisture and static stability of the boundary layer). Little change to the prediction scores indicates any appropriate combination of variables that measure saturation, turbulence, and near-surface cooling can be used. The remaining false-alarm periods are manually assessed, identifying the lack of spatio–temporal information (such as the temporal evolution of the local conditions and the advective history of the airmass) as the ultimate limiting factor in the methodology’s predictive capabilities. Future observational studies are recommended that investigate the near-surface evolution of fog and the role of non-local heterogeneity on fog formation.

## Introduction

We use the diagnostic observational fog-prediction method described by Menut et al. (2014) to predict fog events over a 5-year period at the Cabauw Experimental Site for Atmospheric Research (CESAR) in the Netherlands. Our goal is to identify where the method is limited, why it is limited, and how it may be further improved, particularly with respect to reducing the overall number of false alarms. We test different forecast lead times from 1–6 h, along with different optimization schemes and predictive variables. We further look to understand the underlying reasons for the occurrence of false alarms in the prediction: are they due to an incomplete representation of fog-formation processes, or due to limitations of the methodology itself, which uses only local information in both space and time?

Fog is a meteorological phenomenon that is particularly common in the Netherlands where high humidity and a distinct seasonal cycle provide favourable conditions for its formation. The reduction in visibility that accompanies fog makes it a hazard for navigation, and can be a major cause of motor-vehicle accidents (Bartok et al. 2012) as well as a disruption to maritime shipping operations (e.g., Alpert and Feit 1990; Fu et al. 2010). Fog disruptions to air travel can also have a high economic impact (e.g., Fabbian et al. 2007; Gultepe et al. 2007; Stolaki et al. 2012), with more time needed between plane takeoffs and landings causing delays, as well as the need to cancel flights.

Fog forms when water vapour in the air condenses to form droplets, leading to a reduction in visibility as increasing numbers of water droplets block the optical path via the scattering and absorption of light. Regional and application-based definitions of fog vary; however, for consistency with previous studies (Menut et al. 2014; Román-Cascón et al. 2016a), we use the accepted meteorological definition of fog, i.e., when visibility is below 1 km at the surface (NOAA 2005).

There are several types of fog, and while each is the result of the basic process of droplet formation, the differences in formation conditions and environments are used to distinguish them. Our focus is on radiation fog since this is a common fog type in the Netherlands (e.g., Duynkerke 1999), and its primarily localized nature makes it easier to study the dynamics involved. Radiation fog typically forms at night under calm, clear conditions as the result of radiative cooling of the surface due to an imbalance in the net radiation and subsequent saturation of moist air.

The study of fog has a long history (for a detailed review, see Gultepe et al. 2007), with considerable investigation into the key processes for fog formation and the ability of different methods to predict fog. For example, Duynkerke (1991, 1999) investigated fog in the Netherlands, identifying conditions favourable for the formation of radiation fog (such as the need for a calm, stable boundary layer), as well as the need for reliable physical parametrizations in numerical models. The simulation of fog formation, for instance, is particularly sensitive to the surface roughness, requiring careful attention in models. More recently, Maronga and Bosveld (2017) investigated the sensitivity of a radiation-fog event’s life cycle to various parameters through sensitivity tests and idealized simulations using the Paralellized Large-Eddy Simulation Model (PALM; Maronga et al. 2015). They found the timing of fog onset to be sensitive to turbulent mixing, cold-air advection, and soil temperature, with overall duration and intensity of the fog event additionally sensitive to soil moisture. Further international studies at a range of sites include: the ParisFog experiments (Haeffelin et al. 2010) regarding fog in a semi-urban area around Paris, France; and the Fog Remote Sensing and Modelling (FRAM) project in Canada (Gultepe and Milbrandt 2007), among many others (e.g., Fabbian et al. 2007; Tardif and Rasmussen 2007; Bartok et al. 2012; Boneh et al. 2015; Huang and Chen 2016).

While fog has been studied for many years and it is of clear societal relevance, the accurate prediction of fog events remains elusive (Steeneveld et al. 2015). In part, this is due to the complex interplay between many different processes during formation, and the need to capture not only large-scale dynamics (such as wind speed and the synoptic conditions), but also small-scale processes such as droplet microphysics (e.g., Gultepe et al. 2007). Numerical models are often unable to capture the timing and duration of fog events (e.g., Steeneveld et al. 2015; Román-Cascón et al. 2016a, b). In many cases, this is the result of deficiencies in subgrid-scale parametrizations (Steeneveld et al. 2015), as well as the vertical and horizontal resolutions being too coarse to fully model the necessary scales of relevance and also heterogeneity (e.g., Tardif 2007; Bergot et al. 2015; Philip et al. 2016; Maronga and Bosveld 2017). A further challenge for numerical simulations is in deriving estimates of visibility. Both liquid-water content and droplet-number concentration are needed to accurately estimate visibility in models; often, however, only the droplet-number concentration is included (Gultepe et al. 2006, 2007). At the same time, the relationship between droplet properties and visibility is complex and dependent on whether a fog layer is growing or dissipating (Boers et al. 2013). As a result, a combination of process-based numerical forecasts and a rule-based fog diagnosis may be useful (Zhou et al. 2012).

The prediction of fog from observations dates back at least one century, with Taylor (1917) outlining a simple method for nocturnal fog predition in England based on observations at 2000 local time the evening before. The simple methodology–comparing wind speed and humidity to empirical threshold functions–resulted in a hit rate of roughly 80%, and a false-alarm rate of around 50%. Recently, Menut et al. (2014) developed a similarly straightforward statistical approach with which to forecast radiation-fog events at the ParisFog site using observations of key variables (such as relative humidity) and comparing them to threshold values that indicate the onset of fog in the coming 6 h. Their method was subsequently applied by Román-Cascón et al. (2016a) at the Spanish Research Centre for the Lower Atmosphere (CIBA) and at the CESAR facility. In both studies, the statistical method proved successful in capturing at least 90% of all fog cases analyzed; however, the high rate of correct prediction is still accompanied by a correspondingly high rate of false alarms: almost 40%. It is this method outlined by Menut et al. (2014), hereafter referred to as the M14 method, which we also use to predict fog events at Cabauw and assess the impact of different optimization schemes and the role of different factors leading to false alarms.

While the M14 method has been previously well-documented at different sites, including Cabauw, the reasons for false-alarm occurrence–and correspondingly, means of reducing false alarms–have not been reported in great detail. We look to fill this gap by investigating the role of various forecast windows on the overall predictability, comparing the performance at the suggested lead time of up to 6 h to shorter lead times of 3 and 1 h, in order to assess whether the loss in lead time is compensated by a significant improvement in forecast skill. We also test the ability of different optimization schemes to improve performance, by accepting increased risk (reduced identification of events) in order to increase the confidence of the predictions (reduced false alarms), tuning the methodology for different needs and uses. Further, the potential of other predictive variables (particularly the inclusion of vertical information as opposed to purely near-surface observations) is investigated to determine whether a key process has been ignored in the formulation. Finally, we focus on the reasons why false alarms occur when using the methodology. In part this is related to the search for a previously ignored factor in fog formation. It is also aimed at assessing the impact that the method’s lack of spatio–temporal information has on the prediction; for example, its inability to account for temporal evolution of the system and the advective history of the airmass.

A brief introduction to the CESAR facility follows, with an overview of the M14 methodology and optimization schemes in Sect. 2. The results of applying the methodology with different lead times and optimization schemes are shown in Sect. 3, followed by an analysis of the remaining false alarms in Sect. 4. Finally, a discussion follows in Sect. 5.

## Observations and Methodology

### The CESAR Facility and Observational Data

The CESAR facility (e.g., Monna and Bosveld 2013) is located near Lopik in the province of Utrecht, the Netherlands (51.971$^\circ$N, 4.927$^\circ$E). It is operated by the Royal Netherlands Meteorological Institute (KNMI) and a consortium of research institutes and universities. The site is surrounded by predominantly agricultural fields and small waterways, and is in fairly homogeneously flat terrain, broken occasionally by small villages. The nearest major city is Utrecht, located roughly 20 km to the north-east, with the industrial city of Rotterdam approximately 35 km to the west-south-west. Amsterdam, The Hague and the North Sea are located roughly 50 km to the north and west, respectively. The water table is maintained approximately 1 m below the surface, except during periods of heavy or extended rainfall.

The primary feature of the CESAR facility is the 213-m tall instrument mast, which, in addition to other instruments around the site, including a series of smaller masts and an automatic weather station, measures the vertical profiles of various meteorological variables including temperature, wind speed, and visibility. Data from the site are publicly and freely available through the CESAR website: www.cesar-database.nl.

Visibility–which we use as our primary indicator of fog–is measured at the site with Biral SWS-100 forward-scatter visibility sensors at seven heights: 2, 10, 20, 40, 80, 140, and 200 m. The visibility range of the sensors is 10 m to 20 km. Air temperature and dewpoint temperature are also measured at these seven heights (replacing the measurement at 2 m with one at 1.5 m), with air temperature additionally measured at 0.1 m. Relative humidity is calculated at seven heights from both temperatures, and wind speed and direction are measured along the mast at six heights: 10 m and above. Net radiation is measured as the total (longwave and shortwave) upwelling and downwelling radiation at 1.5-m height. For other variables, the reader is encouraged to visit the CESAR website and accompanying documentation. We use the 10-min averaged observations.

We restrict our analysis to the 5 years of 2012–2016 (inclusive) when visibility data are available at all seven heights at the tower location. An independent fog event is classified as when conditions are foggy (visibility < 1 km) at the 2-m level for at least 50 min out of every 1 h, and separated from a previous event by at least 2 h as in Román-Cascón et al. (2016a). Of all 254 independent fog events between 2012–2016, the most common type of fog at the CESAR facility according to the classification algorithm of Tardif and Rasmussen (2007) was radiation fog, making up roughly 63% of all events. Twenty percent of the events could not be classified with the algorithm and may include further radiative events. These and the non-radiative events are not considered in this analysis. It should be noted that the classification is for the CESAR facility alone, and is likely different for other locations around the country, particularly at the coast.

**Fig. 1:**
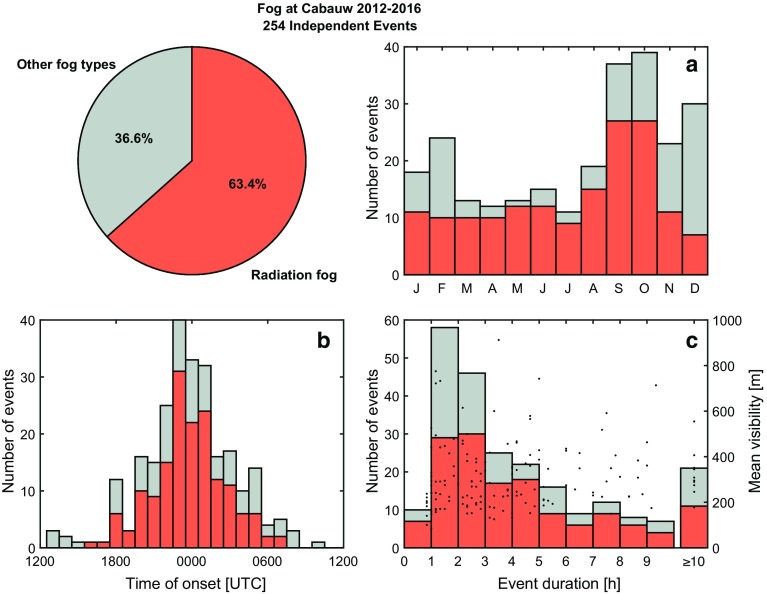
Fog at the CESAR facility between 2012–2016. Out of 254 independent events, radiation-fog events contribute the greatest portion. **a** Total number of fog events per month, **b** time of onset of fog events, and **c** duration of fog events by type with the dots indicating the mean visibility for radiative events

Fog at the CESAR facility occurs throughout the year, with the greatest number of all fog events occurring in autumn (Fig. 1a). Due to the nature of formation, the radiative events form mostly in the middle of the night (Fig. 1b), when radiative cooling has led to a significant reduction in temperature at the surface. With a typical duration of around 1–3 h (Fig. 1c), only a few events persist beyond sunrise. For most fog events the mean visibility for the duration of the event is below 500 m (Fig. 1c) regardless of the duration, with the minimum visibility as low as tens of metres.

### Prediction of Fog from Observations

**Fig. 2:**
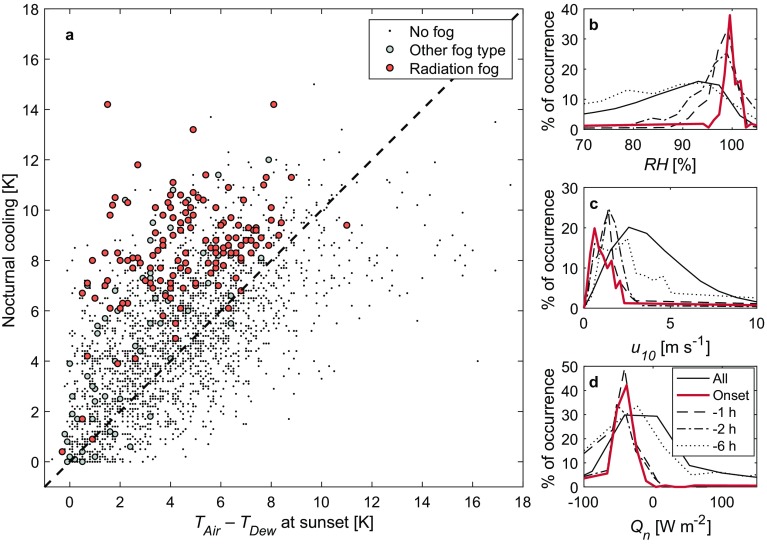
**a** Amount of cooling at 1.5 m over a given night (temperature sunset minus minimum nocturnal temperature) compared to the amount of cooling needed to reach saturation at sunset. Nights with radiation fog events are in orange, nights with other fog types are grey. Nights on which no fog was observed are represented by the black dots. The dashed line indicates the 1:1 equivalency, above which cooling is sufficient to reach saturation and fog events are expected to occur. **b**–**d** Probability density functions of relative humidity at 1.5 m, 10-m wind speed, and net radiation, respectively, up to 6 h before the onset of radiation-fog events. The solid black lines indicate the overall distributions in the data

In the most naïve sense, radiation fog forms when the air cools to saturation (the air temperature is equal to the dewpoint temperature; a relative humidity of 100%). Given that, the simplest prediction of radiation fog would be to compare the air temperature at a given time to the dewpoint temperature (how much cooling is needed for fog to occur), and then predict the amount of cooling that will occur. If the predicted cooling is greater than the cooling required, fog would be expected to form. However, Fig. 2a shows that, for the 1.5-m level at sunset, while it may be a necessary condition that the air temperature must reach (or be very close to) the dewpoint temperature, it is not a sufficient condition for fog formation. Therefore, such a simple prediction cannot be reliably employed.

The conditions before the onset of the radiative events are fairly consistent, however, pointing toward the possibility of prediction based on observations and providing the foundation for statistical–observational methods, such as by Taylor (1917) and Menut et al. (2014). In part, this is due to the classification of events using the Tardif and Rasmussen (2007) algorithm, which identifies radiation fog based on low wind speeds and clear skies; however, it also points to the underlying dynamical processes under which fog occurs. For example, the probability density functions in Fig. 2b–d show almost all of the radiation-fog events occur when the net radiation is strongly negative, wind speed is low, relative humidity is high, and the boundary layer is stable, with the distributions becomming increasingly distinct toward the time of onset. Relative humidity, in particular, exceeds the 95-percentile of all observed values as onset approaches. Likewise, calm conditions and strong radiative cooling are preferred for radiation fog.

While not as distinct as the approach to fog onset, the conditions before dissipation return to the underlying distribution as dissipation approaches (not shown). For example, the wind speed increases and the temperature inversion weakens. All of this is generally initiated by an increase in net radiation (a warming) at the surface, usually as the result of sunrise, though also from the formation and/or passage of clouds.

### M14 Methodology

**Fig. 3:**
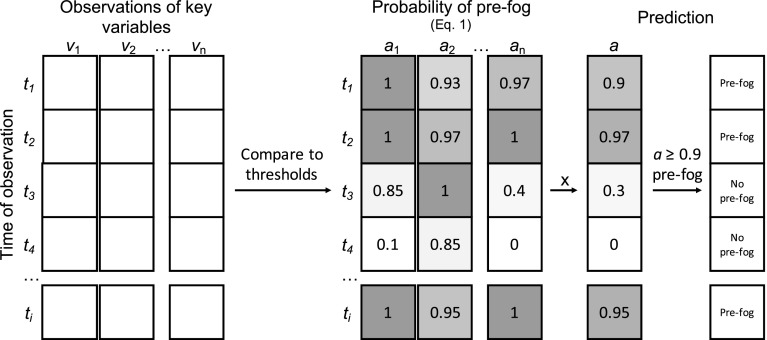
Overview of the M14 method

We investigated strategies for reducing false alarms in observational fog prediction using the M14 methodology. We tested shortened forecast lead times, along with a range of additional predictive variables beyond the original methodology. This section briefly outlines the steps employed by the M14 method, with the reader directed to Menut et al. (2014) for more detail on its development. Given that the conditions preceding radiation-fog events are, in general, distinct, the M14 method assigns a probability of pre-fog conditions based on how close current observations are to the fog-favourable conditions, which is then used to diagnose the likelihood of fog forming within the next 6 h (Fig. 3).

Menut et al. (2014) identified four key variables for the diagnosis of pre-fog conditions: relative humidity (*RH*), net radiation ($Q_n$), wind speed at 10-m height ($u_{10}$), and the overall trend in air temperature from 3 h prior ($\varDelta {}T^{3~h}_{1.5}$). They then define thresholds for each variable (*v*) for which fog conditions are favourable. If the threshold ($v_T$) is met for a given variable, its prediction score $a_v = 1$. If it is not met, the probability is calculated from a tapered normal distribution, allowing for conditions that are close to favourable to still influence the prediction score positively,

1 $$\begin{aligned} a_v = {\left\{ \begin{array}{ll} 1 &{} \text {if threshold met} \\ \frac{1}{\sqrt{2\pi {}\sigma {}_v}}\text {exp} \left[ \frac{-(v-v_T)^2}{2\sigma {}_v}\right] &{} \text {otherwise} \end{array}\right. }, \end{aligned}$$

where $\sigma {}_v$ is the standard deviation of the observed variable, *v*. The overall probability of pre-fog is then calculated as the product of each individual prediction score at each timestep for the variables considered,

2 $$\begin{aligned} a = \prod _{v=1}^{n_v}a_v . \end{aligned}$$

If $a \ge 0.9$, then pre-fog is diagnosed and fog is expected in the next 6 h. This is predominantly an engineering aproach, rather than a physically robust approach (as the properties are not independent and the real likelihood of fog is not necessarily a strict multiplication), but it does provide a means of estimating the statistical likelihood of fog formation based on the observed variables.

The thresholds determined by Menut et al. (2014) for the Paris site are presented in Table 1, along with site-specific thresholds for the CESAR facility as determined by Román-Cascón et al. (2016a) (identified as RC16). RC16-H is the threshold set found to achieve the highest hit rate, and RC16-F is the result of minimizing the false-alarm rate, both over the period from 2008–2011. It should be noted that RC16-F is inherently stricter than RC16-H as the aim is to exclude the highest proportion of false alarms.

**Table 1:** The M14 method thresholds ($v_T$ in Eq. 1) as defined by Menut et al. (2014) for the ParisFog site, and the thresholds for the CESAR facility to achieve maximum hit rate or minimum false-alarm rate from Román-Cascón et al. (2016a)

Variable	Thresholds		
	M14	RC16-H	RC16-F
*RH* (%) $\ge$	90	88	98
$Q_n$ (W m$^{-2}$) $\le$	$-\,10$	5	$-\,20$
$u_{10}$ (m s$^{-1}$) $\le$	3	4	1.5
$\varDelta {}T^{3~h}_{1.5}$ (K) $\le$	$-\,0.5$	0	$-\,1.5$

In Sect. 3, the results of applying the M14 method at Cabauw using the original thresholds are presented for a prediction window of 6 h as well as shorter windows of 3 and 1 h, with a diagnosis made at each 10-min observation. From the PDFs in Fig. 2b-d, the shorter windows should lead to improved performance of the method; however, naturally, the degree to which the method is improved may not be sufficient to outweigh the loss of lead time. Only nocturnal radiation-fog events lasting more than 2 h are considered (as with Román-Cascón et al. 2016a).

Further to the proposed combination of four variables, we investigated a total of 31 alternative variable combinations in addition to, and in replacement of, the original variables. The additional criteria include thresholds on such variables as the friction velocity ($u_*$) and the trend in visibility over the past 1 h as proposed by Román-Cascón et al. (2016a), as well as those that include not only surface observations, but vertical information as well (such as the temperature inversion between the surface and aloft, and the relative humidity at 10-m height). We also considered, inter alia, the comparision of the observed heat flux to the maximum sustainable heat flux of van de Wiel et al. (2012). While microphysical properties (such as aerosol concentration and type) are important for fog formation, the possibility of a microphysical predictive variable was not investigated due to the lack of appropriate observational data. For a complete list of the combinations assessed, see Table 4 in Appendix 1.

### Assessment of Prediction Performance

Several indicators of prediction performance can be used, each with their own merits. We use the hit rate (*HR*), and false-alarm rate (*FA*), which were applied by Menut et al. (2014) and Román-Cascón et al. (2016a) as their primary indicators of prediction performance. Hit rate and false-alarm rate are based on the comparison of predictions and actual fog occurrence on a timestep-by-timestep basis. If (as per Table 2) the number of correct predictions is *h* (pre-fog diagnosed and occurs; hits) and the total number of missed predictions is *m* (fog occurs that is not diagnosed), then the hit rate (in percent) is simply the ratio of correct predictions to the total number of actual pre-fog occurrences (both those correctly diagnosed and those missed),

3 $$\begin{aligned} HR = 100\left( \frac{h}{h+m}\right) . \end{aligned}$$

Correspondingly, where the number of incorrect predictions is *f* (pre-fog diagnosed but not observed; false alarms), and the number of correct non-predictions is *c* (no pre-fog diagnosed and no fog in reality), the false-alarm rate is the number of false alarms divided by the total number of non-fog cases in reality ($f+c$),

4 $$\begin{aligned} FA = 100\left( \frac{f}{f+c}\right) . \end{aligned}$$

**Table 2:** Forecast parameters for calculating *HR* (Eq. 3) and *FA* (Eq. 4)

	Pre-fog obs.	No Pre-fog obs.
Pre-fog pred.	Hit (h)	False alarm (f)
No pre-fog pred.	Miss (m)	Correct clear (c)

In practice, *HR* and *FA* values can be misleading, with the score dependent on the overall length of the time series. For example, if very few fog events are observed, then a single hit carries significant weight when assessing the hit rate. Likewise, the false-alarm rate is very low if the time series is long due to the large denominator. As such, their values must always be taken into consideration with other factors such as the length of the time series.

### Optimization for Desired Outcomes and Acceptable Risk

A perfect prediction with $$ HR = 100\%$$ and $$ FA = 0$$, while ideal, is far from realistic. As such, the performance of a forecast must be judged not only on the number of correct and incorrect predictions made, but also on the needs of the user and stakeholders. Just as in numerical modelling where efficiency of calculation must be balanced with resolution demands, so too is there a trade-off between accurate foreasting and permissible levels of risk in a forecast.

It is conceivable that for some scenarios overprediction is desired (i.e., there can be little to no risk that an event be missed). Conversely, there may be some acceptable level of missed events when operations or needs are able to adjust. For example, this might occur at an airport where procedures are in place to adapt for inclement weather and the financial loss of altering schedules for an incorrect fog forecast could be greater than for delays caused by an occasional unforeseen fog event.

To this end, we tested different optimization schemes in order to demonstrate and evaluate the ability of the approach to be tuned for different needs. Using a simple evolutionary algorithm, the first 3 years of the dataset (2012–2014) are used to attain the desired performance by optimizing the thresholds of the different variables, and then the optimized thresholds are subsequently applied to the following 2 years (2015–2016). The optimization criteria are as follows:

1. max*HR*: Thresholds are optimized to obtain the maximum hit rate possible.
2. max$\varDelta HR _{95}$: Some risk is tolerated, with thresholds optimized to first obtain $HR \ge 95\%$, after which the value of *FA* is minimized.
3. max$\varDelta$: Thresholds are optimized to achieve the largest difference between the hit rate and false-alarm rate.
4. max$\varDelta FA ^5$: Very little risk is tolerated, with thresholds optimized to achieve $FA \le 5\%$, after which the value of *HR* is maximized.

Section 3.1 shows the impact that different optimization schemes have on the forecast scores when applied to the M14 method at Cabauw.

## Performance of the M14 Method at Cabauw

In this section, the results of applying the M14 method at Cabauw for 2015–2016 are presented for lead times up to 1, 3, and 6 h. The performance of the optimized criteria is presented first for the original four variables proposed by Menut et al. (2014), followed by the results of using the different variable combinations described in Appendix 1.

### Optimized Performance for Different Lead Times

**Fig. 4:**
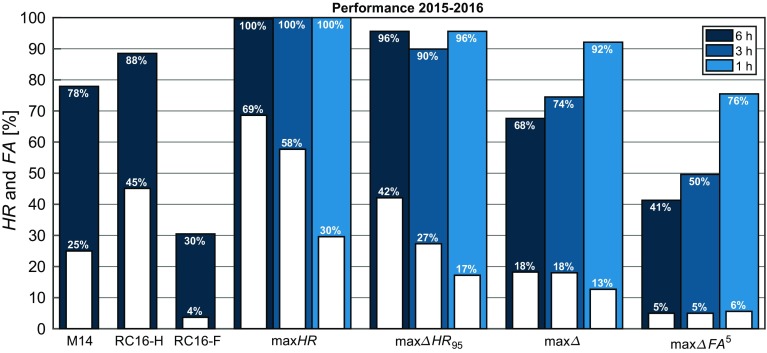
Hit rate (blue) and false-alarm rate (white) for pre-fog diagnosis between 2015–2016 using the original thresholds in Table 1, and thresholds optimized at 6, 3, and 1-h lead times according to the criteria described in Sect. 2.5. The optimized thresholds are in Table 3

We applied the M14 method as outlined in Sect. 2.3 using the newly optimized threshold values as described in Sect. 2.5. First, we optimized the thresholds using the data from 2012–2014, subsequently using these optimized thresholds to generate forecasts for 2015–2016. The results are presented for lead times of 1, 3, and 6 h (Fig. 4). The performance of the original thresholds from Menut et al. (2014) and Román-Cascón et al. (2016a) at a lead time of 6 h are presented for reference. The optimized threshold values for each of the four variables of *RH*, $Q_n$, $\varDelta {}T^{3~h}_{1.5}$, and $u_{10}$ for the different schemes are in Table 3.

**Table 3:** Optimized thresholds according to the different criteria in Sect. 2.5 for the three lead times tested

Variable	Optimized thresholds			
	max*HR*	max$\varDelta HR _{95}$	max$\varDelta$	max$\varDelta FA ^{5}$
1 h				
*RH* (%) $\ge$	89.4	93.1	94.1	96.6
$Q_n$ (W m$^{-2}$) $\le$	6	$-$ 9	$-$ 19	$-$ 19
$u_{10}$ (m s$^{-1}$) $\le$	2.2	2.0	2.0	1.4
$\varDelta {}T^{3 h}_{1.5}$ (K) $\le$	3.8	1.6	3.6	$-$ 1.1
3 h				
*RH* (%) $\ge$	76.0	80.1	81.1	91.6
$Q_n$ (W m$^{-2}$) $\le$	14	1	15	5
$u_{10}$ (m s$^{-1}$) $\le$	3.4	2.0	1.9	1.3
$\varDelta {}T^{3 h}_{1.5}$ (K) $\le$	2.3	$-$ 0.5	$-$ 1.3	$-$ 2.3
6 h				
*RH* (%) $\ge$	73.0	72.7	75.0	91.4
$Q_n$ (W m$^{-2}$) $\le$	8	3	15	$-$ 25
$u_{10}$ (m s$^{-1}$) $\le$	4.3	2.4	2.0	1.4
$\varDelta {}T^{3 h}_{1.5}$ (K) $\le$	7.1	2.9	$-$ 1.5	$-$ 2.1

The max$\varDelta HR _{95}$ and max$\varDelta FA ^5$ criteria show similar performance to their RC16 counterparts, as expected since the optimization constraints are essentially the same. At 6 h, the other optimizations, max*HR* and max$\varDelta$, do not perform well, with either a false-alarm rate that is far too high (70%) despite $HR = 100\%$, or an overall higher false-alarm rate (18%) for a relatively low hit rate (just under 70%).

Re-optimizing with decreasing lead time, however, the optimized thresholds show a distinct score improvement over their equivalent 6-h predictions. In the case of the thresholds optimized to obtain a high hit rate (max*HR* and max$\varDelta HR _{95}$ ), the false-alarm rate decreases by 10% by shortening to a 3-h window, and up to 40% for the 1-h window. Likewise, the hit rate increases significantly for the optimizations that favour a lower false-alarm rate (max$\varDelta$ and max$\varDelta FA ^5$). Again, there is a roughly 10% improvement at 3 h, and between 25–35% for the 1-h window.

It should be noted that the improvement in scores is not simply due to a change in denominator in Eqs. 3 and 4 (e.g., larger denominator for *HR*—more pre-fog, and smaller denominator for *FA*—less non-pre-fog, at 6 h than at 1 h), as this changes the score between just 0.1–5%. With the shorter lead time, all optimizations result in either > 90% of all events being captured with the false-alarm rate between 13–30%. Or, conversely, very few events falsely forecast, with the hit rate over 75%. Compared to the original RC16-H and RC16-F thresholds, and the newly optimized thresholds at 6 h, this is a significant improvement.

**Fig. 5:**
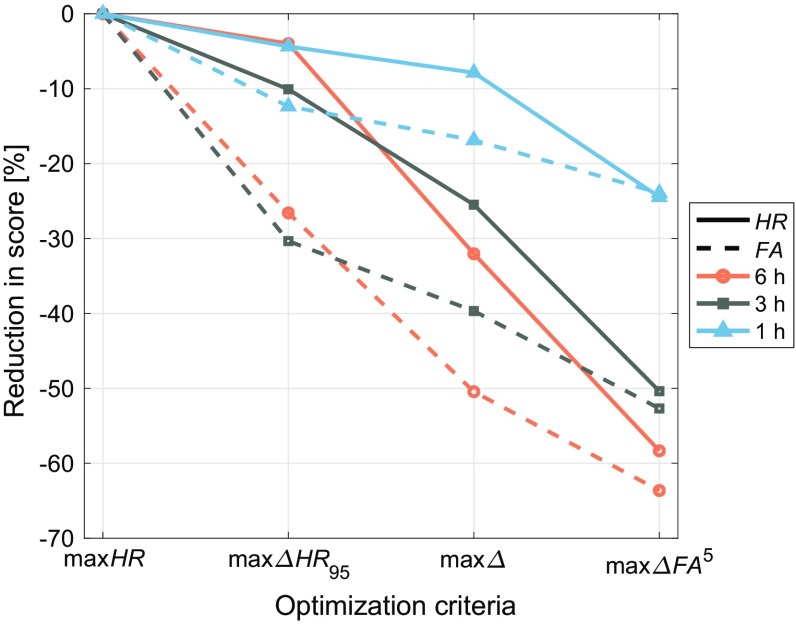
Reduction in hit rate (solid) and false-alarm rate (dashed) relative to the max*HR* criteria for increasing strictness of the thresholds (increased risk for increased confidence; left to right)

Comparing the different optimization schemes for a given time window, it is apparent that *FA* values are initially more sensitive to stricter thresholds than *HR* values (by allowing for an incremental increase of risk, a larger increase in confidence is obtained; Fig. 5). In particular, accepting just a small decrease in the hit rate (max*HR* to max$\varDelta HR _{95}$), the false-alarm rate is immediately reduced by 10–30% (particularly for larger lead times). This suggests there are significantly more cases where it is marginally a no-fog forecast than there are cases for a marginal fog forecast. Interestingly, however, the max$\varDelta FA ^5$ thresholds lead to roughly the same reduction in the hit rate as in the false-alarm rate from the max*HR* optimization criterion (a drop of 20% at 1 h and 60% at 6 h).

The greatest compromise between a high hit rate and a low false-alarm rate is provided by the max$\varDelta$ optimization criterion at 1 h, providing $HR >~90\%$, and a false-alarm rate of just over 13%. It is, however, only a marginal improvement over the max$\varDelta HR _{95}$ optimization criterion, which is the best performer over all lead times. With a hit rate of 96% and false-alarm rate of 42%, it is the equivalent to the RC16-H threshold at 6 h. As the forecast window is reduced to 1 h, the hit rate is maintained while the false-alarm rate is reduced to just under 20%, which, while still far from ideal, is a reduction of over 50%.

Although significant improvements in predictive score can be achieved through employing shorter lead times and optimizing the thresholds to suit different needs, it is not possible to achieve a perfect score ($$ HR = 100\%$$ and $$ FA = 0$$). In the following sections, we investigate whether optimal performance can conceivably be achieved with the M14 method. First, alternative parameters are assessed (Sect. 3.2) in order to identify any potentially missing (local) factors in the methodology. The remaining reasons for false alarms are subsequently investigated in Sect. 4.

### Other Variable Combinations

We tested a total of 31 variable combinations to determine whether additional or alternative parameters could be used to better diagnose radiation fog than using the original four parameters of *RH*, $Q_n$, $u_{10}$, and $\varDelta {}T^{3~h}_{1.5}$ proposed by Menut et al. (2014). An overview of the different combinations is given in Appendix 1.

**Fig. 6:**
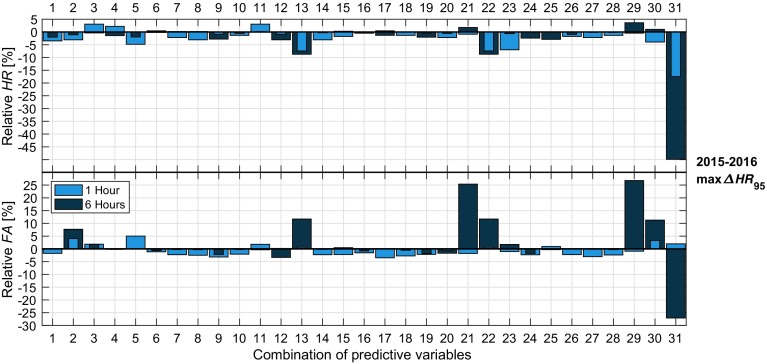
Relative hit rate (upper) and false-alarm rate (lower) for the max$$\varDelta HR _{95}$$ pre-fog diagnosis between 2015–2016 at 1 h and 6 h for the different combinations of variables compared to the classical combination. The *y*-axis shows the difference between the scores with the alternative combination and the classical scores (i.e., relative $$ HR = HR _{alt.} - HR _{class.}$$). The numbers on the *x*-axis correspond to the different combinations in Table 4

Overall, none of the 31 other variable combinations resulted in a significant improvement in the scores; irrespective of the chosen variables, the optimization schemes result in nearly identical performance for most alternative combinations (Fig. 6). It is also not consistent for different optimizations and different lead times whether or not a combination leads to an improvement in the overall score.

For example, consider the inclusion of the modified fog-stability index (*FSI*; see Table 4 #17 for the relation)—either in addition to the original variables, or in replacement of *RH*—at 10 and 200 m ($FSI_{10}$ and $FSI_{200}$; 17–21 in Fig. 6). At best, the false-alarm rate is reduced by around 5%. While only a relatively small percentage, the 5% reduction is a significant decrease given the overall number of observations (a 5% reduction is equivalent to almost 2000 fewer false alarms). However, this is only the case for the max*HR* and max$\varDelta HR _{95}$ criteria at 1 h, with no significant change at the other lead times or for the other optimization criteria.

Interestingly, dewpoint depression, which is a key component of the fog-stability index, does not have a significant impact on prediction scores (8 and 9 in Fig. 6), nor does including other forms of vertical information, such as the temperature inversion (14–16). Likewise, inclusion of further temporal information, such as the trend in relative humidity (24), makes no difference to the scores. The combinations proposed by Román-Cascón et al. (2016a) also do not lead to a significant change. In fact, they are slightly detrimental to the overall optimized scores at 1 h , with the prediction using $u_{*}$ in replacement of $u_{10}$ (2) leading to a decrease in hit rate and increased false-alarm rate.

Several criteria sets are notably worse in performance than the classical prediction at 6 h . Sets 13 (adding $RH_{10}$), 21 (replacing $u_{10}$ with $FSI_{10}$), 22 (including time to sunrise), and 29–31 (only predicting on $FSI_{10}$, $FSI_{200}$, or visibility) all result in either increased a false-alarm rate or decreased hit rate at 6 h (between 10–50%). At 1 h, however, all but the prediction on visibility alone (#31) result in similar scores compared to the original four-variable combination. Even still, visibility is not an entirely meaningless predictor at 1 h, with a hit rate as high as 80%.

## Reasons for Remaining False Alarms

Even with the optimization of the thresholds and reducing the lead time to 1 h, there is a maximum performance for the prediction methodology. A hit rate of 100% is possible, but at the cost of a false-alarm rate of roughly 20%, and even then, at only a 1-h lead time. Likewise, low false-alarm rates are attainable, but at significant cost to the hit rate. Here, we aim to identify the cause of the false alarms for the max$\varDelta HR _{95}$-optimized thresholds at 1-h and 6-h lead times.

**Fig. 7:**
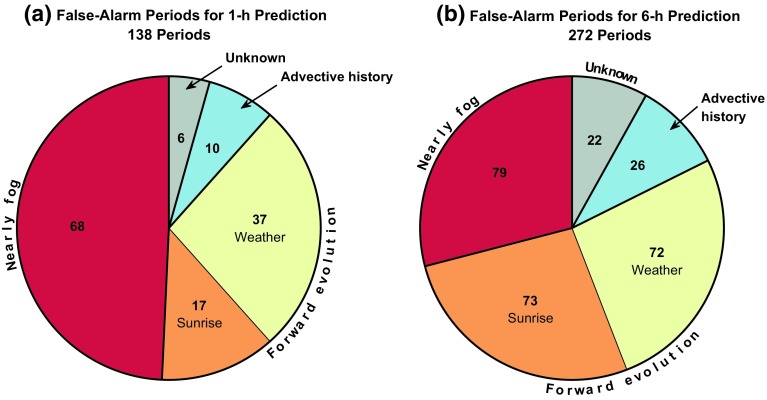
Total number of false-alarm periods manually classified according to the primary reason for the false alarm at both **a** 1-h and **b** 6-h prediction windows using the max$\varDelta HR _{95}$-optimized thresholds

Each false-alarm period was classified manually according to the observations during the period (Fig. 7). A false-alarm period is defined here as being continuously falsely diagnosed as pre-fog for at least 1 h in order to eliminate sporadic diagnoses. Periods where two or more variables are at the threshold level are also excluded from the analysis in order to avoid the influence of thresholds that are too relaxed. In total, there were 138 and 272 false-alarm periods analyzed for the 1 and 6-h prediction windows, respectively.

A large portion of the false-alarm periods (roughly 50% and 30% of the periods at 1 h and 6 h, respectively) are cases where the conditions are very close to being foggy, but not quite when observed at the 2-m level. These include cases where the prediction is made too early (i.e. that fog does form, but after the prediction window) and situations where the visibility is below 1 km (or close to 1 km) for most of the false-alarm period, but never reaches the conditions required to be classified as a fog event. Note too that radiation fog may be very shallow. It is therefore possible that there is a shallow fog layer that does meet these conditions, but is not observed at 2-m height. It is important to note that the overall scores change little when a different, more relaxed, definition is applied for the visibility (e.g., a threshold of 1.6 km as in Tardif and Rasmussen 2007).

The remaining false-alarm periods are divided according to three main categories:

1. Forward Evolution of the System The M14 method is purely diagnostic based on recent observations (zero-dimensional in time), with no information about the future evolution of the system. This includes something as simple as the rising of the sun (more significant for the 6-h prediction) as well as a change in synoptic weather conditions. In total 54 out of 138 of the false-alarm periods for the 1-h prediction were due to changes to the system after the time the prediction was made, and 145 out of 272 of false-alarm periods for the 6-h prediction.
2. Advective History of the Airmass The method assumes that the local conditions are also representative of the upwind conditions (spatial influence), with the advection of an airmass with different properties (e.g., warmer or drier air of urban origin) making the conditions unfavourable for fog at a later time. 10 out of 138 false-alarm periods at 1 h, and 26 of the 272 periods at 6 h, were diagnosed as due to advection of unfavourable air properties (identified by changes in the temperature and/or humidity without a distinct change in wind speed, radiation, or turbulent mixing).
3. Unidentifiable from the Observations Alone Not all false alarms were obviously attributable to any cause based on the observations. These cases may include the exclusion of a key process in the prediction as well as the possibility of missed fog due to observational limitations (false false alarms; e.g., fog that is present, but too shallow to be captured at the lowest measurement level of 2-m height). This accounts for just 6 false-alarm periods for the 1-h lead time and 22 for the 6-h lead time.

**Fig. 8:**
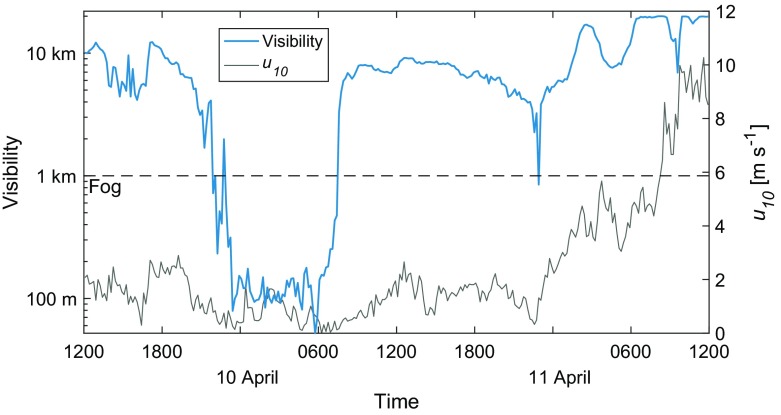
Visibility and wind speed for 48 h starting at 1200 UTC on 9 April 2015. Radiation fog formed on the first night, but not the second due to the increase in wind speed around 2300 UTC

An example of a false alarm caused by a change in the synoptic conditions is in Fig. 8, which shows visibility and $u_{10}$ on the the nights of 9–10 and 10–11 April 2015. The two nights are neighbours in Fig. 2a with seasonal effects of temperature, solar radiation, and length of night (among other factors) roughly identical for the two days: both nights cooled around 15 $^\circ$C from sunset, with an initial dewpoint depression of around 10 $^\circ$C. The nocturnal evolution of the temperature inversion was approximately the same at the start of these “twin” nights, with low wind speeds (< 2 m s$^{-1}$), high relative humidity (increasing from 80% at sunset up to 100%), strong surface cooling ($\varDelta {}T_{1.5}^{3~h}~<~-4$ K), clear skies ($Q_n~<~-45$ W m$^{-2}$), and overall ideal conditions for radiation fog formation. Fog did indeed form on 9 April and last until sunrise on 10 April. However, just as fog was again about to form on 10 April, the wind speed increased as the result of a frontal system, making the conditions no longer favourable for fog formation. A diagnosis made up to 2300 UTC on both nights would, however, predict fog in the coming hours due to lack of information about the change in the system to come.

Including additional predictive variables that incorporate temporal information, however, does not lead to a reduction in the false-alarm rate. This was considered as part of the search for an additional predictive metric (Sect. 3.2), through including both the time before sunrise and the trend in atmospheric pressure, among others, with little change in the predictive scores.

**Fig. 9:**
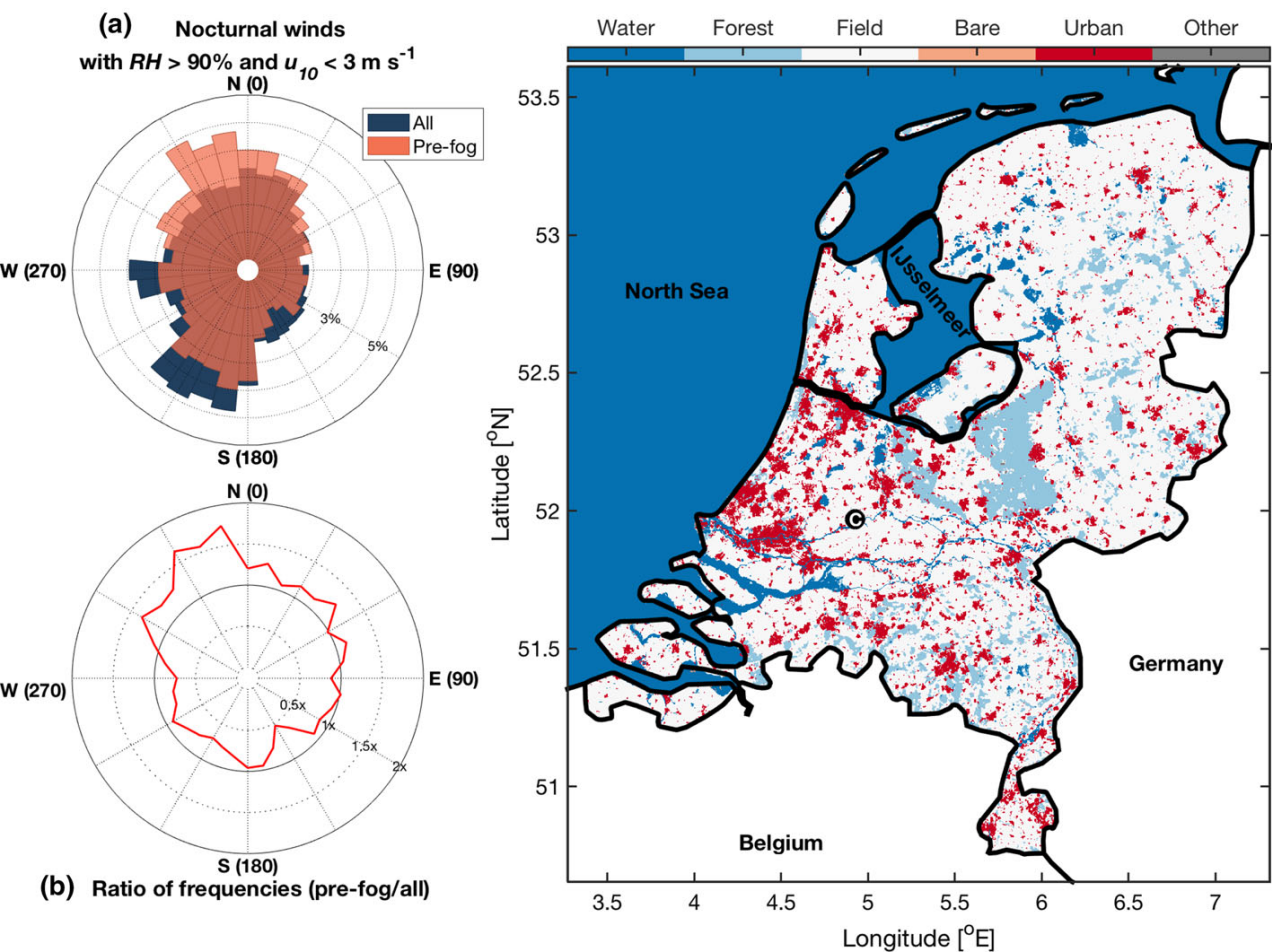
300-m land use in the Netherlands in 2015 with **a** the probability density function of observed nocturnal wind direction for conditions where $RH~>~90\%$ and $u_{10}~<~3$ m s$^{-1}$, as well as the subset that occurs up to 6 h before a radiation-fog event. **b** The ratio of the probability density functions in (**a**). The CESAR facility is marked on the map with the letter C in the black circle. Land use is from the ESA Climate Change Initiative (Hollmann et al. 2013) land-cover dataset (accessible here: http://maps.elie.ucl.ac.be/CCI/viewer/index.php), broadly grouped into six categories

The advection of warmer, drier air accounts for only a small fraction of the false-alarm periods assessed (10 and 26 periods for the 1-h and 6-h predictions, respectively), but is likely a contributing factor to the overall predictability and climatology of fog at Cabauw. The effect is most apparent when the nocturnal wind direction at Cabauw is considered (Fig. 9). The dominant ($>~$50%) 10-m nocturnal wind at the CESAR facility is from the south to south-west and is the same for periods with high relative humidity ($>~$90%) and low wind speed ($<\,$3 m s$^{-1}$). If there is no directional influence on the formation of fog at Cabauw, the distribution of winds preceeding fog events should be nearly identical. However, the distribution of winds for the subset of observations up to 6 h before a radiation-fog event is from predominantly the northward direction, with a correponding reduction in other directions, particularly from the south to south-west. In fact, situations where the flow originates from the north-west to north lead to fog almost twice as frequently as would be expected from the underlying distribution.

While it is difficult to use point observations to assign a definitive reason for the reduction of the south to south-west peak in the distribution of observed winds that preceed radiation-fog events, we hypothesize that it is due to the history of an airmass prior to its arrival at Cabauw. When air travels from the north, it passes over predominantly flat agricultural land for several kilometres, in a region that is very similar to the Cabauw site, and much available moisture in the form of lakes and the North Sea and IJsselmeer. In contrast, the air that travels from the south to south-west must pass over more industrial regions (including the port city of Rotterdam) as well as over an embankment just south of the CESAR facility (leading to enhanced turbulent flow), and is also more likely continental air from the south.

This hypothesis is supported by the fact that the nights on which the wind is blowing from the south to south-west quadrant cool on average 2 $^\circ$C less than from the other directions. In many cases, this influence is excluded in the prediction with the threshold on $\varDelta {}T_{1.5}^{3~h}$, but not if a change in wind direction occurs throughout the night. It should be noted that this difference in distributions is not related to seasonal variability in the wind direction (even in autumn when fog is most common, the wind is predominantly from the south to south-west).

## Discussion

### Application of the M14 Method

We applied the M14 method at the CESAR facility to assess its predictive performance for the years 2015–2016 at both different lead times, and with different optimization schemes. In all, the methodology, comparing observations of specific variables to pre-determined thresholds, is straightforward to implement at the Cabauw site, with the results here showing the method provides valuable insight into whether or not a radiation-fog event is expected to occur.

Even at 6-h lead times, the two intermediate optimization schemes (max$\varDelta$, and max$\varDelta HR _{95}$) lead to hit rates of approximately 70–95% and false-alarm rates of just 20–40% (Fig. 4). These optimizations favour a greater difference between hit rate and false-alarm rate, rather than necessarily attempting to maximize or minimize one of them, which would result in correspondingly high false-alarm rates (max*HR*) or low hit rates (max$\varDelta FA ^5$) at 6 h. As the forecast window is decreased, however, the predictive capabilities of the M14 method are significantly enhanced for all optimizations. Optimizing to achieve the best hit rate (max*HR*), for example, a hit rate of 100% is achieved with a 6-h prediction window, but with a false-alarm rate of 70%. With a reduced forecast lead time of 1 h, the false-alarm rate drops to just 30%. Overall, this significant improvement in the forecast score is encouraging and, depending on the needs of the user, may serve to offset the reduced lead time.

A similar effect is seen when the optimization constraints are made stricter (Fig. 5). Whether a 6, 3, or 1-h lead time, accepting a small degree of risk in the form of a reduced hit rate immediately results in a significant increase in confidence of the prediction (i.e., reduced false-alarm rate). For example, the false-alarm rate is reduced by two to five times as much as the hit rate when comparing the results using the max*HR* and the max$\varDelta HR _{95}$ criteria. The relative reduction in *FA* and *HR* values is related to the parameter space of the observed variables for fog and non-fog events. Initially, as the thresholds become stricter, proportionally more non-fog events are excluded than fog events, resulting in the larger reduction in *FA* values than *HR* values. Eventually, the thresholds enter into the parameter space where most fog events occur, with further strictening of the thresholds leading to the exclusion of a large number of fog events and the subsequent reduction of the *HR* value.

It should be noted, however, that even with a reduction in the false-alarm rate, there is still a significant amount of false predictions. This is hidden in the calculation of *FA* values, with the total number of false alarms divided by the total number of observations which are not pre-fog (see Eq. 4). Although a false-alarm rate of 13% from the max$\varDelta$ optimization at 1 h, for example, appears reasonable, there are almost seven times as many non-fog obervations as there are pre-fog obervations, meaning that roughly 50% of all predictions of fog are false. This is an improvement on the 6-h prediction, but is still a concern when applying the methodology.

In an effort to reduce the false-alarm rate further, we tested 31 additional variable combinations as alternatives to the original combination of variables proposed by Menut et al. (2014). None of the combinations, which include soil heat flux and vertical information, lead to any significant improvement in the prediction scores, or in fact any consistent change at different lead times or for different optimizations. This is most likely due to the interdependence of many of the variables (for example, dewpoint depression and relative humidity are essentially measuring the same thing), suggesting that any combination that measures the saturation, the atmospheric stability, and the cooling are sufficient for the methodology. This is a helpful insight when considering the application of the methodology at different sites. Although the original four variables chosen are commonly measured at meteorological stations, the fact that they can be interchanged with complementary observations means the method is even more portable.

The amount and type of aerosols present could not be considered in the analysis. Such data are limited in temporal coverage at Cabauw, and only at a height of 60 m, which does not correspond to the surface observations here. As such, direct analysis during the fog events considered here is not tractable. Aerosols are critical for fog formation, however, with hygroscopic particles acting as condensation nuclei for water droplets. Without them, much higher saturation relative humidities must be achieved. Boers et al. (2015) also indicated that decreasing hygroscopicity of aerosols in the Netherlands is correlated with a decrease in the occurrence of fog events in the Netherlands. It may be that some false-alarm periods correspond with times of low aerosol content, or the presence of aerosols with reduced hygroscopicity. It would be enlightening to perform a dedicated observation campaign where particle counts and hygroscopicity can be measured in order to determine what impact they have on the prediction results.

Although the events are filtered such that they last for at least 2 h (and are therefore fully established as in Román-Cascón et al. 2016a), it should be noted that the method does not explicitly distinguish between the severity of different fog events (e.g., in terms of the depth of the fog layer, or the minimum visibility). The hazard presented by a fog event is due to the reduction in visibility, with different modes of transportation able to better overcome this than others. Aircraft, for example, are higher off the ground when taxiing, and fly through low cloud at takeoff and landing, which is very different than a car driving down a highway. This implies that what is dangerous on highways is very different than what is dangerous at airports. Further study is needed to determine the factors that result in thicker and deeper fog layers.

### Reasons for False Alarms

One of our aims was to identify the reasons for false alarms when employing the M14 method. In Sect. 4, false-alarm periods lasting at least 1 h when using the max$\varDelta HR _{95}$ criteria were manually assessed in order to identify a reason for the lack of radiation-fog formation despite the threshold criteria being met. In total, 138 periods were identified for the 1-h, and 272 periods for the 6-h prediction windows (Fig. 7). Ultimately, it appears it is the lack of spatio–temporal information included in the method that limits its performance.

A large portion of the false-alarm periods are when the conditions are very close to, but not quite, fog events according to the definitions employed here (including predictions that are made too early, or when conditions are not sustained long enough to be classified as an event). The higher occurrence of “nearly fog” cases at the 1-h prediction window than the 6-h window is due to the conditions under which radiation fog occurs being much more definite at a shorter lead time (corresponding with the decrease in spatio–temporal false alarms). These borderline cases are less serious false alarms as they still represent periods of risky, reduced visibility conditions, in contrast to cases which are fully clear in spite of the pre-fog diagnosis. It would be interesting to explore such borderline situations further, where sensitivity to environmental parameters is likely high, but that is beyond the scope of our study.

While near-fog cases are mostly the result of the definition of fog events assessed, the remaining false-alarm periods are primarily due to the lack of temporal and spatial information encoded in the methodology. The M14 method is almost entirely zero-dimensional: a prediction is made at a single time and location based on the observations at that given time and location. As such, the method does not consider the possibility of the system evolving in time due to spatial and temporal influences. Three primary reasons for these non-local spatio-temporal causes for false alarms were addressed here: sunrise, synoptic weather/internal dynamics, and, related, the advective history of the airmass.

The so-called forward influences (in time) of sunrise and weather influences result in unfavourable local conditions after the prediction has been made (e.g., increased net radiation or wind speed as in Fig. 8). The influence of sunrise is particularly significant for the 6-h forecast window, where 6 h may be much longer than half the night. As such, there is a greater likelihood that the sun will rise before fog conditions are reached. Including time to sunrise as a predictive variable does not, however, lead to improved prediction scores (#22 in Fig. 6), instead resulting in increased *FA* values, and decreased *HR* values. At both the 1-h and 6-h prediction windows, the influence of weather (such as clouds and fronts) on the false alarms is approximately the same, indicative of the more random passage of such systems, as opposed to the very definite rising of the sun.

In addition to assuming a temporal stationarity, the method also inherently (and by necessity) assumes that the observations are representative of not only the current local conditions, but also the upwind conditions. However, if the upwind conditions are drastically different than the local conditions say, for example, that there is a city upwind where the air is warmer and relative humidity lower, then the predictions made at a given location may not be valid. Even though wind speeds are relatively low in the event of radiation-fog formation, an airmass propagating at a wind speed of just 3 m s$^{-1}$ can travel approximately 10 km in the space of 1 h. That means any prediction made at a given time could potentially be on a parcel of air with entirely different properties than the upwind parcel that will be at the prediction location just a short time later.

Figure 9 shows that nocturnal flow with high relative humidity ($RH~>~90$%) and low wind speed ($u_{10}~<~3$ m s$^{-1}$) is more likely to lead to fog if they originate from the north-west to north than any other direction. This is in direct variation to the underlying distribution of winds, which are more common from the south to south-west. Including wind direction as a further predictive variable, however, would not lead to an improvement in prediction scores at the CESAR facility. As the radiation-fog events are not restricted to a single well-defined direction, but merely indicate a preference for a wind direction, to do so would result in a large number of missed pre-fog cases. While upwind influences do not result in a significant portion of false-alarm periods diagnosed here for Cabauw (Fig. 7), the analysis indicates that the broader land surface heterogeneity can play a role in the formation of localized fog events. This suggests that the localized picture of the factors influencing radiation fog is not entirely accurate. It is conceivable that in regions with larger variability than Cabauw, such as near cities or airports, such an effect may be further enhanced.

It is also possible that the relationship of wind direction is not entirely landscape-based, but related to overall circulation patterns. In particular, the sea-breeze transition described by Arrillaga et al. (2018) could be a compounding factor, with moist, aerosol-rich air coming from the sea leading to the enhancement of fog likelihood. A larger climatology of events would be needed to study such cases, however, as the number of events identified by Arrillaga et al. (2018) is small. Further analysis is beyond the scope of this work.

In some cases, a definitive reason for a false-alarm period could not be easily determined from the observations alone. However, we hypothesize two reasons for such cases which are related to observational restrictions. The first is that there is a critical variable which has not been considered. As shown in in Sect. 3.2, 31 variable combinations were tested in addition to the original four key variables proposed by Menut et al. (2014). These resulted in little change in overall optimized performance of the prediction method, but it is possible that some further factor is not considered. Aerosols, or the lack thereof, as discussed above, could prove important for marginal cases where conditions appear right for fog formation, but cannot form without appropriate condensation nuclei.

**Fig. 10:**
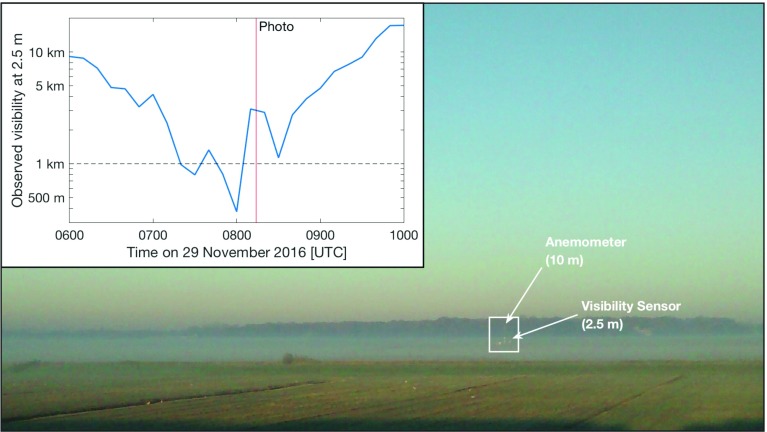
A dissipating shallow fog layer on the morning of 29 November 2016. A weather station can be seen (in the white rectangle), with the visibility sensor above the shallow fog layer and therefore not observing foggy conditions at the time of the photo (inset). The distance to the weather station is approximately 700 m, and 1.3 km to the tree line from the camera

It is also possible that there is fog present, but it is too shallow, or too patchy, to be captured by the single-point observations at 2-m height. It is not uncommon, for example, to see shallow fog layers (see Fig. 10), or to drive along a road at night and suddenly encouter a patch of fog drifting across the road. Such conditions have also been demonstrated numerically, with high-resolution simulations of fog at the Charles de Gaulle Airport in Paris demonstrating the role heterogeneous land surfaces play in the resulting formation of heterogeneous fog (Philip et al. 2016). In this case, it is not necessarily a false alarm in the strict sense as there is still fog present, just not observed (a false false alarm). Of course, there is no way to investigate the full influence of such effects on the predictions made here with the available point observations, but it does pose interesting questions that should be considered in future observational campaigns and analysis, with high-resolution observations in this missed layer. While such events are perhaps not as hazardous as deep, thick fog cases, the presence even of shallow or sparse fog can be dangerous, particularly for automobile traffic. Likewise, understanding cases of shallow or patchy fogs, and whether or not they continue to develop into thicker, deeper fog layers is important for understanding the full lifecycle and evolution of fog events.

Although the false-alarm periods are presented as being due to the influence of one dominant factor, it should be noted that they are in reality the result of concurrent factors. For example, it will take longer for fog to form with reduced aerosol content and when air upwind is warmer, resulting in slower cooling locally. Given the extended period of time before sufficient cooling and condensation has occurred for fog to form, a frontal system or sunrise will have been more likely to break down the favourable conditions for formation. As such, the identification of these factors may be viewed as the final factor in leading to the false alarm (e.g., if there was no sunrise there could still be fog, just later in time), and seen as more of a qualitative assessment rather than robust quantitative analysis.

### Practical Implications

#### Implications for Numerical Simulations

Our findings were not tested against numerical models, however, the results do present challenges for simulations of fog. For example, we show that models will need to not only capture the localized conditions (and accurately model the local surface characteristics), but also factors on a range of scales. For example, upwind heterogeneity in surface properties may be important for the formation of local fog, but how much of this heterogeneity needs to be considered within a model? This is unclear and will likely depend on the regional variability of the simulation domain. At the same time, large-scale weather patterns have to be included. This conflict of scales, from highly localized to synoptic, may be best achieved through a nesting approach. Such an approach could, for example, be employed for specific regions of interest such as airports, where numerical studies have shown high horizontal and vertical resolution are needed to capture the influence of land-surface heterogeneity induced by buildings and runways (Bergot et al. 2015; Philip et al. 2016). Yet, nesting remains a challenge. In fact, Steeneveld et al. (2015) demonstrated nesting could potentially prove destructive to fog simulation, rather than constructive.

Both Menut et al. (2014) and Román-Cascón et al. (2016a) also assessed the performance of the M14 method using numerial output. In both cases, the model did not accurately capture the necessary conditions needed for fog to form, resulting in poor predictive capabilities. This is due to the difficulty in correctly capturing the local characteristics within the model (such as surface coupling, turbulent mixing, etc.), which is a common problem when performing fog simulations (e.g., Steeneveld et al. 2015) and a factor which has been shown important for the lifecycle of fog in sensitivity studies (Maronga and Bosveld 2017). It is possible that including more information on the local land surface and improving horizontal resolution may improve results.

Vertical resolution of the models is also important. For example, Tardif (2007) and Maronga and Bosveld (2017) showed that sufficient resolution is needed to properly capture the evolution of fog. This is also important for capturing the initial growth phase of fog from the surface. The lack of near-surface observations, however, could make validation of such models difficult.

#### Operational Application of the M14 Method?

The M14 method is useful as a diagnostic tool, however, its limitations as discussed here must always be considered. As such, it is best viewed as a supplementary tool, rather than the sole tool for fog prediction.

Nevertheless, assessing the performance of different optimization schemes at different lead times provides insight into how the M14 method can be used in an operational setting. Depending on the needs of the user, a first prediction can be made at 6 h, which is useful for preparedness, but may not yet be critical. At this point where operations are not yet being adjusted, for example, overprediction may in fact be useful. With subsequently decreasing lead time, the confidence of the prediction, regardless of the optimization method, improves significantly.

An example of this application using the max$\varDelta HR _{95}$ threshold set is as follows. With a 6-h window, a diagnosis is made based on current observations that is expected to capture 96% of the events, though with a known over-prediction with *FA* = 42% (Fig. 4). Those in charge of adjusting operations or producing warnings are now aware that an event is possible, but may not need to react. A shorter forecast window is then used to update the initial prediction, maintaining roughly the same hit rate, but now with increased confidence; the false-alarm rate is reduced by 15% at 3 h. By the 1-h prediction window, the prediction is over twice as certain as it was at 6 h, and operations can now be adjusted and warnings issued accordingly, in conjunction with other information received through the use of a numerical forecast and the forecasters’ experience and knowledge.

## Conclusions

Using observations from the CESAR facility in the Netherlands, we assessed the performance of the statistical method for diagnosing the onset of radiation-fog events developed by Menut et al. (2014). Our goals were: to determine the influence of different lead times on the forecast, to test the role of different optimization schemes and the potential for improved prediction through consideration of additional variables, and to identify reasons for false alarms.

Even at a lead time of up to 6 h, the M14 method is a useful diagnostic tool that can provide a hit rate of over 90% and a false-alarm rate under 40%. With decreasing lead time, we have shown that the method becomes increasingly more accurate for prediction, with the false-alarm rate being cut in half or the hit rate doubled. The significantly improved accuracy may help to offset the reduced benefit from shortened lead times depending on the needs of the user. In any case, the performance at reduced lead times encourages its use as a diagnostic now-casting and monitoring tool over short time periods.

We tested four different optimization schemes focused on different needs that balance confidence and acceptable risk, demonstrating the versatility of the M14 method to be tuned and applied for different applications. Accepting just a slight decrease in the hit rate (increased risk) leads to a significant reduction in the false-alarm rate (increased confidence). This is an important and beneficial implication for implementation of the method in an operational setting.

In assessing the reasons for false alarms, 45–65% were attributed to either the forward temporal evolution of the system (changes in the weather conditions, for example), or the advection of warmer, drier air from upwind. Both spatio–temporal factors cannot be fully eliminated with the purely local method (in both space and time) and must be considered when employing the methodology. This also presents a potential challenge for numerical simulation of fog events, with a competing need to capture large scale influences (such as synoptic weather) and increasingly localized influences (e.g., land surface coupling and heterogeneity). In an effort to reduce the spatio–temporal false alarms using the M14 method, it would be interesting to assess the impact of supplementing the observations with numerical output from a synoptic weather model, or even a simple statistical regression model; both of which may help to provide an indication of the large-scale system evolution.

Further study should include seeking to understand the role of vertical growth of the fog layer both at the near-surface and toward deeper, thicker fogs; the influence of non-local heterogeneity on local fog formation; and the role aerosols play in determining whether or not fog events will occur. Further, the methodology should continue to be evaluated at different sites where local land use and climatology are different than at Cabauw. Finally, it would also be beneficial to explore similar diagnostic methodologies related to other fog types beyond radiation fog, which is the exclusive focus of the M14 methodology.

## Appendix 1: Other Variable Combinations

In addition to the combination of four variables proposed by Menut et al. (2014)–here referred to as “Classic”–we tested a further 31 combinations where additional variables are either added to the set of predictors, or used in replacement of the original variables. They are summarized in Table 4.

**Table 4:** The 31 other combinations of variables tested beyond the original four variables of Menut et al. (2014))

Identifier	Description
Additional near-surface information	
1. $+u_*$	Friction velocity must be low (no turbulence) for fog formation (as proposed by Román-Cascón et al. 2016a)
2. $-u_{10} +u_*$	Replace 10-m wind speed with the friction velocity (as proposed by Román-Cascón et al. 2016a)
3. $+G_0$	Soil heat flux. Should be low or negative
4. $+|H_0|$	Absolute value of the sensible heat flux. Should be close to 0. (no turbulence)
5. $+[MSHF-H_0]$	Compare the *maximum sustainable heat flux* of van de Wiel et al. (2012) to the sensible heat flux. Should be small positive, or negative
6. $+$min$u_{10}$	A minimum threshold on the wind speed (air should not be completely stagnant)
7. $+$min$Q_n$	A minimum threshold on the net radiation. (Fig. 2d shows fog does not form when net radiation is too low, likely indicative of the available moisture)
8. $+[T-T_D]$	Dewpoint depression (difference between air and dewpoint temperatures at 1.5 m). Should be close to 0 or negative
9. $-RH +[T-T_D]$	Replace relative humidity with the dewpoint depression
10. $+[T_{1.5}-T_{0.1}]$	Difference in air temperature at 1.5 and 0.1 m. Should not be too great or dew/frost formation may remove moisture and/or fog will be too shallow
11. $+[T_{1.5}-T_{Soil}]$	Difference in air temperature at 1.5 m and soil temperature at $-0.02$ m
12. $+P_{atm}$	Include atmospheric pressure at the surface. High pressure favourable
Additional vertical information	
13. $+RH_{10}$	The 10-m relative humidity cannot be too low, otherwise entrainment of dry air from above could disrupt fog formation
14. $+\varDelta \varTheta _{10}$	Include the temperature inversion between 10 and 1.5 m and 200 and 1.5 m. A stronger inversion is favourable due to increased stability of the surface layer
15. $+\varDelta \varTheta _{200}$	
16. $-\varDelta T_{1.5}^{3~h} +\varDelta \varTheta _{200}$	Replace the temperature trend with the temperature inversion between 200 and 1.5 m
17. $+FSI_{10}$	A modified *fog stability index* calculated at 10 and 200 m height (as opposed to the 850 hPa level; Holtslag et al. 2010): $FSI_z = 2(T-T_{D})+2(T-T_{z})+u_{z}$. Low values are favourable, with $\le$31 typically indicative of fog at 850 hPa
18. $+FSI_{200}$	
19. $-RH +FSI_{10}$	Replace relative humidity with $FSI_{10}$ and $FSI_{200}$
20. $-RH +FSI_{200}$	
21. $-u_{10} +FSI{10}$	Replace 10-m wind speed with modified $FSI_{10}$
Additional temporal information	
22. $+[t_{sun}-t]$	Time to sunrise. Should not be too close to rising sun, otherwise conditions will no longer be fog-favourble (increased radiation, warming, decreased relative humidity)
23. Mean 1 h	Apply thresholds on the mean value of the classic variables over the past 1 h
24. $+\varDelta RH_{1.5}^{3~h}$	Analogous to $\varDelta T^{3~h}_{1.5}$, the relative humidity at 1.5 m must not be decreasing significantly over the previous 3 h
25. $+\varDelta V_{2}^{1 h}$	The visibility at 2 m must be decreasing over the previous 1 h (as proposed by Román-Cascón et al. 2016a)
26. $+\varDelta P_{atm}^{3 h}$	Change in atmospheric pressure over 3 h. Should not decrease significantly (worsening of synoptic conditions)
27. $+Precip_{24}$	Total precipitation in previous 24 or 48 h. Indicator of soil moisture
28. $+Precip_{48}$	
Single variables	
29. Only $FSI_{10}$	Only predict on $FSI_{10}$ or $FSI_{200}$, which include dew point depression and wind speed
30. Only $FSI_{200}$	
31. Only $V_{2}$	Only predict based on the visibility at 2 m

$+$ Denotes an addition to the classical variables while − indicates one of the original variables is replaced (except when in [ ])
